# Ethnic predisposition, risk factors and breast cancer presentation; a 10-year data. Single centered prospective cohort study from Karachi

**DOI:** 10.1016/j.amsu.2022.104612

**Published:** 2022-09-09

**Authors:** Naheed Sultan, Saeed Ahmed Memon, Mehwish Mooghal, Shah Wali, Wajiha Khan, Hina Tahseen, Memuna Khan, Daniyal Monis

**Affiliations:** a Department of Surgery, PNS Shifa Hospital Affiliated with Bahria University of Health Sciences, DHA Phase 2, Sailors Street, Karachi, Pakistan; b Department of Surgery, CMH Rawalpindi, Rawalpindi, Pakistan; c Department of Surgery, Dow University of Health Sciences, Baba-e-Urdu Road, Karachi, Pakistan

**Keywords:** Breast cancer susceptibility, Breast cancer, Familial breast cancer, Right sided, Family history, brca1 brca2, Ethnicity

## Abstract

**Background:**

Breast cancer, a leading cause of mortality among females, has been the center of research for many decades. Work is in progress to advance the research worldwide and in our region. This study is conducted to look into regional ethical predilection/age, clinical presentation/stage, pathological subtypes and risk factors of BC among patients of Karachi, with the aim of proposing a ground in our policy making regarding protocol setting for screening and management of BC patients.

**Methods:**

A prospective cohort study started at public Hospital, Karachi from 2010 to 2020.500 females with histo-pathologically proven BC selected. History, clinical examination, radiological and histo-pathological data retrieved; data regarding age, ethnicity, family history, parity, marriage/menopause, stage/lump size/symptoms were filled on pro-forma. Primary outcomes were age, ethnicity, family history, stage/histological type and menopausal status of our cohort while secondary outcomes were parity, marriage, symptoms and lump size/site. Data analyzed using SPSS in ranges and percentages.

**Results:**

Among different ethnicities, Makrani were the most affected(34%). Majority were premenopausal females ≤50yrs (78%). Infiltrating ductal carcinoma (88.8%) was the commonest subtype. Family history was positive in few (5.8%). Parity and marital status had no effect on our population. Breast lump (88%) was the commonest presenting symptom and 51% of our patients had the right side involved. Upper outer quadrant (51%) was the most involved quadrant and the majority (46%) were stage II.

**Conclusions:**

Age of presentation is around a decade earlier in our region, with women of Makrani descent more prone to develop BC. 2/3rd of patients were premenopausal, with lump breast as primary complaint. Majority of patients presented in stage-II. Results of age and racial predilection in our population suggest us to concentrate future research more on genetic profiling so we incorporate the results to devise population specific protocols with reference to age, presentation, BC type, ethnicity & risk factors.

Record submitted retrospectively at ClinicalTrials.govt on 09-07-2022 ***NCT05458570***.

## Highlights

• Regional ethical predilection, diagnosis age, clinical presentation, pathological subtypes and risk factors of BC among patients of Karachi, Pakistan.
• Need of institutional/national level guidelines for breast cancer, specific to our population parameters.
• Age of presentation is around a decade earlier in our population as compare to the West.
• Screening age criteria specific to our population should be proposed and implemented.
• Women of Makrani decent are the most affected ethnic group from Karachi, followed by Memons.

## Introduction

Breast cancer is now identified as the most common cancer of females with incidence rate of 43.1/100,000 and 25.1% reported cancer incidence out of all known cancers [1]. Almost 12.9% of all cancer deaths are attributable to BC, second only to lung cancer [1]. Returned surveys from GLOBOCON 2012 yielded region wise highest incidence rates of 91.6% and 91.1% from northern America and western Europe, while lowest rates of 26.8% and 27% from middle Africa and eastern Asia. The incidence rate of BC among US women is 1 in 8, while that of Asian women is 1 in 35 [[2], [3], [4]]. However, recent trends are showing steady rise in incidence of BC in Asian habitants also [1]. Over 50% of BC incidence is now reported from developed world with increase of incidence rate to 69/100,000; now it stands as the commonest cancer among women in this continent also [[4], [5], [6]]. Pakistan's name is now quoted among the top countries with highest reported mortality rates besides Fiji, Bahamas & Nigeria [1]. Putting forward the trends of BC in Pakistan, it is now the most frequently diagnosed cancer in females with 2.5 times higher rates than the neighboring countries with highest reported death rate [[6], [7], [8]]. However, the epidemiology of breast cancer in Pakistan is difficult to describe accurately, mainly due to the lack of proper reporting and tumor registry system of Pakistan [5,6].

With rising incidence, genetic factors in BC have always been the focus of research which contribute to about 15% of all BCs. The known incidence is 1 in 10 among overall reported BC cases, while in young BC patients the genetic susceptibility is reported as 1 in 8.5 [9]. The most important established factor that increases the relative risk by 1.1–3.6 times is having BC in one or more first degree relatives [9,10]. Majority of inherited breast cancer is associated with BRCA1 and BRCA2 [9,10]. Lifetime risk of developing BC is as high as 80% in BRCA1 gene along with development of ovarian cancer [[9], [10], [11]]. In Pakistan, the prevalence and penetrance estimates for BC suggest that dominant BRCA1 and BRCA2 mutations are significant contributors to breast and ovarian cancer in our population [12]. However, the likely association between parental consanguinity and BC risk suggests that recessive genes may also play a role in the etiology of BC in young Pakistani women [12].

Similarly pertaining to age, Carol E. et al. reported that 82% of BCs are diagnosed among women ≥50 years of age and around 90% deaths of BC fall into this age group, with mean age at diagnosis of BC as 62 years [13]. This study also commented on different racial groups and prevalence of specific subtypes of BC and presentation among population. Black women were more prone to develop aggressive tumor subtypes and locally advanced stage at presentation, with tumor size ≥ 5 cm, higher mortality as compared to other ethnicities [13]. However we lack data of ethnicity, specific tumor subtypes & size prevalent in our population due to unavailability of proper data reporting system in Pakistan, this makes our treating teams restricted in decision making and incorporating guidelines that are proposed by West.

Other risk factors associated with BC like age at first full term pregnancy(≥30 years), obesity, early menarche(≤12yrs)/late menopause(≥50yrs), upper socioeconomic class, nulliparity, white race and geographical distributions [5]. In a study conducted at the two major cancer hospitals of Pakistan, nulliparity, less number of children, late menopause and positive family history have been found to have a relationship with the occurrence of breast cancer [14]. However, this study only included the women younger than 45 years and the data cannot be applied to the females above 45 years.

The purpose of this study is to find out age at diagnosis, ethnic preferences, histological types and stage of BC on presentation, along with associated risk factors responsible for BC in our population, so the results of the study can be implemented and used in future policy making for breast cancer programs in the country, setting regional screening age criteria for our population, genetic testing criteria and catering any modifiable risk factors positively with mass education programs and help find new research domains which can have significant effects on future breast cancer research in our region.

## Methods

A prospective cohort single-centered study conducted, which included 500 female patients who attended one surgical unit of a tertiary care public hospital Karachi, during the period of 2010–2020. The study was performed in line with the principles of the Declaration of Helsinki and data collection started after taking hospital ethical review board's approval because personal data of patients was used. Its Research Registry UIN is NCT05458570 (https://clinicaltrials.gov/show/NCT05458570). Afterwards, informed written consent was taken from every included patient. Prior to collecting data, all researchers were thoroughly trained with regards to data collection and examination of patients to eliminate observer bias. Non-probability consecutive sampling technique was used. The allotted patients were followed by the same researcher from presentation till the end of follow up to avoid observer bias; through direct patient interaction in OPDs and wards, radiological and histo-pathological results from investigations performed and regular follow up of patients during the complete disease period. The retrieved information was filled in pre-designed pro-forma. Patients were preemptively explained about our reason for collecting data and its implications. The study is reported according to STROCSS 2021 guidelines [15]. Our inclusion criteria was; female sex, age ≥20 years, patients with availability of complete clinical details, biopsy proven breast cancer, no previous history of breast cancer treatment from other centers, all regional ethnicities (residents of Karachi, Sindh only) and all stages of breast cancer. Exclusion criteria of our study was; previously treated breast cancer patients, recurrent BC, female ≤19 years, male/transgender, females coming for treatment from other provinces/foreigners.

Thorough history of all patients obtained including relevant risk factors; i-e age, family history of BC, marital status, age at first born baby, parity, age of menarche/menopause, socio-economic status and ethnicity. Detailed physical examination including clinical presentation of breast lump, its size, side (right/left breast) and site, nipple discharge, nipple retraction, skin involvement, fixity to underlying structures, and lymph node status, was carried out in each patient by a single examiner. Patients presenting with a discrete lump, nipple discharge, nipple changes, skin changes, palpable axillary lymph nodes were subjected to further investigation. Ultrasonography and mammography of the breast, where possible, were performed as initial imaging modality. FNAC/Biopsy was carried out in all patients to confirm the diagnosis of BC, its subtype and receptors status. For staging; x-ray chest, ultrasound abdomen for liver and pelvis/CT chest & abdomen were done on case-based need. Bone scan was performed in only symptomatic cases. After thorough overall assessment, clinical stage of BC was assigned to each patient, and stage-based treatment was carried out in every patient. Patients having localized disease (≤stage 2A) underwent surgical intervention first followed by chemo/radiotherapy, regional disease (≥ stage 2B) had neo-adjuvant followed by surgery, and advanced disease (stage 4) were given palliation [16]. Post operatively detailed histological report of specimen and microscopic involvement of the lymph nodes status were also noted.

Primary outcomes of our cohort were age, ethnicity, family history, stage/histological type and menopausal status while secondary outcomes were parity, marriage, symptoms, lump size/site and socioeconomic status. After collecting the required data on pro forma, it was analyzed using descriptive statistics by SPSS version 23.0 software. With a sample size of 500, co-operation rate was 100% and our confidence interval was 97.5% with 5% margin of error. For quantitative data, mean and standard deviation were calculated. Qualitative results were calculated in percentages and presented in tabular forms.

## Results

Using non-probability consecutive sampling, the data from 500 patients presented to the surgical unit of public sector hospital Karachi was included in study and no withdrawal of patients reported during the time from recruitment till follow up. Over the 10 years period of data collection, out of 500 patients 290 patients belonged to lower middle class (58%), 170 were from lower social class (34%) and only 40 patients were from upper middle/middle class (8%). Ethnicity data showed that Makrani/Balochi were 170/500 (34%), Memon 120/500 (24%), Muhajir 75/500 (15%), Pakhtoon/Hazara 50/500 (10%), Sindhi 45/500 (9%) and Punjabi/others were 40/500 (8%). Mean age of the patients at the time of diagnosis was 46 years (range 20–83 years) with SD ± 15.17, confidence level of 95%, margin of error 46.038 ± 1.33 (±2.89%) with skewness of 0.52142.375(75%) out of 500 patients were less than 50 years and 125(25%) were ≥50 years. Most (82.8%) of the patients were premenopausal and only 17.2% were postmenopausal. Majority 461/500(92.2%) were married. 97(19.4%) were nulliparous, 166(33.2%) of the patients had 1-3 children and 237(47.4)% had more than 3 children. Only few patients had their first baby born after the age of 30 years (24%) while majority (56.6%) had their first child before the age of 30.29(5.8%) out of 500 patients had positive family history of breast cancer. Age of menarche could not be established, as the patients did not remember in most of the cases their exact ages of first cycle. In our study late menopause was defined as those who had menopause after the age of 50yrs which was found to be 6.2%(31/500).

In our study, right sided BC was more common(51%) than the left one (45%) with the bilateral BC reported in very few patients (4%). The data regarding site of breast cancer involvement is presented in Table 1.

**Table 1:** Site of involvement.

S. No	Site of Involvement	No. of Patients	%age	Total patients
1	UOQ^a^	255	51	N = 500
2	Central	120	24	
3	UIQ**	55	11	
4	LIQ^b^	25	5	
5	LOQ^c^	30	6	
6	Whole Breast	15	3	

a Upper Outer Quadrant.

b Lower Inner Quadrant.

c Lower Outer Quadrant About 443(88.6%) patients presented with breast lump, (106)21.2% with nipple discharge, (100)20% with nipple inversion or fixity, skin involvement (tethering & peau-d’-orange) was present in 142(28.4%), skin ulceration in (20)4% of the patients. However, overlapping of two or more symptoms was present on clinical assessment at the time of arrival.

Nearly 265(53%) out of 500 patients had breast lump size ≥5 cm at the time of presentation. 282(56.40%) of the patients had clinically involved lymph nodes on presentation, while that figure increased to (411)82.20% on histological assessment of axillary lymph nodes after surgery.

The data on clinical stage at the time of presentation and histological types of BC is summarized in Table 2, Table 3.

**Table 2:** Stage at presentation.

S. No	Stage	No. of Patients	%age	Total Patients
1	Stage 0	4	0.80	N = 500
2	Stage I	15	3.0	
3	Stage II	230	46.0	
4	Stage III	190	38	
5	Stage IV	61	12.2

**Table 3:** Histopathological types of Breast cancer.

S. No	Histopathological Type	%age	Total Patients
1	Infiltrating Ductal Carcinoma	89.20	N = 500
2	Tubular Carcinoma	4.2	
3	Medullary Carcinoma	1.0	
4	Pagets Disease	2.0	
5	Ductal Carcinoma in Situ	1.0	
6	Poorly differentiated Carcinoma	1.6	
7	Sarcoma	1.0	

## Discussion

BC, the commonest reported malignancy of females overall, as well as in Pakistan; estimated to affect 1 in 8 females during a lifetime [3]. More than 1.2 million women are anticipated to develop BC yearly, according to W.H.O's published statistics [[2], [3], [4]]. The statistics are reportedly rising in Pakistan as well, the incidence is expected to increase by 60.7% by the year 2025 in Karachi only [7]. This incidence rate is surprisingly high as compared to our neighbors with the similar socio-cultural background [8]. Studies from other Asian countries, also reported significant rise of BC in their population. In Singapore, according to the statistics released by National disease registry(2021), between the years 2014 & 2018, 11,232 new cases of BC have been reported and ≥2100 females died of breast cancer, accounting to around 17.3% of cancer related deaths [17,18]. The data also pointed out that it is more prevalent among females from Chinese ethnicity but the worst 5 year survival rate was reported among Malay women(58.5%), with likely younger age at diagnosis, presentation at advanced stages and more aggressive tumor biology in contrast to other ethnic groups of the region[13] [18]. In our study, the highest rates are reported for Makrani/Balochi(34%) women followed by, Memon(24%), Muhajir(15%), Pakhtoon/Hazara(10%), Sindhi(9%) and Punjabi/others(8%) respectively. The likely factors associated with increased incidence among makrani/balochi females which needs to be addressed in future studies, could be their belonging from less privileged areas which contributed to no/little knowledge of the disease at initial stages, genetic factors and consanguineous marriages in the community. The presence of family history of breast cancer doubles the risk of subsequent breast cancer development especially among young women [19]. In our study only 5.8% of the patient had positive family history for BC, our percentage is less as compared to other studies, which reported the risk of BC development can increase up to 19% in patients having positive history of BC in first degree relatives when other risk and demographic factors are adjusted [20]. In an Indian study, family history of breast cancer was found in 20.2% of BC patients presented [21], and amongst them 29% was reported in females younger than 44 years, which is more than the percentages reported in elderly breast cancer patients with family history, depicting association of family inheritance with younger age at presentation. This risk is further increased if there is a history of more than one first degree relatives with BC, younger age at diagnosis of the affected family member and bilateralism of the disease. Probably the reason forwarded was preference of intermarriages among specific communities and ethnicities in this part of the world, which is leading to homogeneity in genetic pool, with increased inheritance of mutated tumor susceptible genes in affected families like BRCA1, BRCA2, TP53 & other emerging recessive genes [21]. S khaliq et al. looked for a link between TP53 gene polymorphism and its predisposition to develop BC in nine major ethic groups from Pakistan. Differences in allele frequencies of 3 polymorphisms were identified in BC patients of different ethnicities. The 16-bp duplication absence was common among Hazara(northern Pakistan), Msp I A1 allele frequency was significant among Makrani people with BC [22]. These results imply that further research work is needed to study the genetic profile of different ethnic groups of Pakistan to look for susceptible genes. Similarly in Japan, they studied the incidence of BC between urban and rural population, their stats showed that the incidence of BC is rising in both rural and urban areas but the anticipated rise is more in urban population which will increase by up to 102.2% by the year 2040, the likely reason could be urbanization of the population; but we failed to study the effect of urbanization of different ethnicities to Karachi, which is the biggest metropolitan city of the country [19].

Age related data from USA indicates that incidence rate are substantially higher for women over 50 years and older compared with women younger than 50 years of age [23]. In contrast Asian results are different, the latest survey from Singapore reveals that 55.6% of the cases of BC involve women below 54 years of age, majority being premenopausal, having stage 2 at the time of initial diagnosis & invasive ductal subtype on histo-pathology results [18]. Our results are comparable to above stated results, with mean age at diagnosis is 46 years and 75% of our patients were less than 50 years, while 2/3rd of the patients were premenopausal. In a similar study from Lahore, their median age of diagnosis was also 45 years [24]. These results from Karachi and Lahore are similar to Malaysia, which reinforces our proposal that the age range of BC in Asia is still less than European countries with likely interplay of genetic and environmental factors which are contributing to early age at diagnosis in our part of world [18,24,26].

In a study including 1482 patients from a South African hospital, they looked for laterality of BC among their subjects. They found that 96.3% patients had unilateral BC while 3.7% had bilateral BC, which is similar to our results of 96% unilateral cases and only 4% presented with bilateral disease. However they reported that left sided breast cancers (55.3%) were more common than right sided, which are contrary to our results where right sided breast cancers(51%) are seen more [27].

Regarding data on stage at the time of diagnosis in Pakistan, Soomro R and et al. reported that the majority of patients present at stage 2 (47.26%) and roughly ≤4% were detected in stage 1. In our study 46% of the cases presented in stage 2 while 38% in stage 3, forming the majority of the cases falling in stage 2 and 3, which is also similar to the study stated above. Their study also reinforced our results of BCs diagnosis at a younger age group in Pakistan, which is at least a decade earlier than the reported age in the west [26].

For symptoms on presentation, Moodley j and et al. stated that breast lump was the most common symptom on presentation (74%) which is also consistent with our results (88.6%), followed by mastalgia, change in breast size, nipple changes and axillary lump [28,29]. Other symptoms reported by our patients were nipple discharge, nipple inversion, skin involvement and skin ulceration in descending order. For breast lump size, a study from Singapore reported that only 11% of their patients presented with lump size of ≥5 cm at the time of first hospital visit for their symptoms, in contrast; our study showed that 53% of our patients presented with lump size of ≥5 cm at their primary visit which is alarming and need to be addressed accordingly [29].

Histology as a prognostic factor has well been documented. Patients with infiltrating ductal carcinoma have poor survival as compared to other histological types. In one of the Indian study the infiltrating ductal carcinoma was found to be the most common i.e. 86%, almost same as in our study (89.2%) [21]. In a series of 369 patients by Sunita Saxena et al. they stated that only sole clinical assessment of lymph nodes under report the stage of BC and after histological confirmation, lymph node involvement in their cases was increased to 80.2% which led to disease upstaging [21]. This is similar to our cohort where 82.20% patient had histological confirmation of lymph node involvement as compared to only 56.6% of patient who had involvement of the lymph nodes on clinical assessment.

For associated risk factors, a study conducted in Madras (India) showed that there was no significant association between age at menarche and BC [25]. Single women had higher risk than married women, and nulliparity was found to be a risk factor in premenopausal women only. The relative risk increased with age of marriage and age at first birth. A three-fold risk noted in both pre and postmenopausal groups if interval between age at first birth and menarche is more than 12 years and in women who attained menopause between the ages of 44–49 [25]. In contrast our results shows poor relationship between being married, as almost 92% of women were married. Majority, 62.8% of BC patients were premenopausal. Nulliparity was also insignificant, as only about 19.4% of the women were nulliparous, while 33.2% had 1-3 children but the majority 47.4% with BC had >3 children. Similar to our results, study including 7663 Malaysian women, did not find any significant association between parity, age of first full term pregnancy and menopausal status; however they reported breastfeeding, soy products intake and active lifestyle as important factors in reducing BC risk [30]. Another study from Pakistan reported no association with age of menarche [14], while in our study age of menarche could not be established as most of the patients did not remember their age at the time of first cycle. However only 24% of the patients had their first-born child after the age of 30 years which reinforces weak association of age at first full term pregnancy and BC development in our population, which is similar to the Malaysian cohort [30].

## Limitation and strengths

Our research also has some grey areas that need to be addressed. We did not categorize urban and rural patient presentations to look for the effect of migration/lifestyle changes on BC in our study. We did not look for prognosis of BC among different ethnic groups of Pakistan, as this information could help us find at-risk ethnicities of our region and broaden future research in the area of genetics for at-risk groups. At the moment we cannot generalize our results because the data is from a single centre only and involves habitants of a single city and surrounding suburbs, however more data is needed from different areas of Pakistan to generalize the results and devise proper screening and management plans specific to our population. We were not able to compare rising or declining trends of BC in our population, because we lack the data retrospectively from our center. We faced recall bias from patients during history taking, specifically when inquiring about age of menarche. To keep the confirmation bias minimal, we kept few interviewers and examiners throughout, who were seeing allotted patients from start till the end of follow-up. Researchers were trained before the start of study to keep the bias minimal. Our long study period helped us recruit a good sample size. We specifically looked for ethnic predilection of BC in our cohort, that was not done previously from our area or less data is available, therefore our study can provide a new ground to look for ethnicity preferences of BC in our region and study genetic profiles of those ethnicities.

## Conclusions

The age of presentation in our cohort is a decade early, so we can say that the age of BC in our country is earlier than the rest of the world and therefore, two third of the patients were premenopausal also. Age parameters and ethnic preference of Makrani/Baloch women has made us think to widen our horizon of research in the field of genetics, so the prevalent genes in our population can be studied in detail and age/population specific screening/management protocols can be devised. Furthermore, mass education programs are needed in the country to educate about the disease and role of consanguinity among communities regarding inheritance of defective genes & genetic pooling of BC genes.

## Funding

The authors declare that no funds, grants, or other support were received during the preparation of this manuscript.

## Author contributions

“All authors contributed to the study conception and design. Material preparation, data collection and analysis were performed by [Dr. Naheed Sultan], [Dr.Saeed Ahmed Memon] and [Dr Mehwish Mooghal]. The first draft of the manuscript was written by [Dr. Saeed Ahmed, Dr. Mehwish Mooghal, Dr Shah Wali, Dr. Wajiha Khan, Dr. Hina Azfar, Dr. Memuna Khan] and all authors commented on previous versions of the manuscript. All authors read and approved the final manuscript.”

## Provenance and peer review

Not commissioned, externally peer-reviewed.

## Ethical approval

Not applicable.

## Please state any sources of funding for your research

This research has not been sponsored and funded by any group or a person.

## Author contribution

• Naheed Sultan: Contribution to the concept/design; acquisition, analysis, and interpretation of data, drafting and revision of article for the content and final approval before submission and accountable for all aspects of work.
• Saeed Ahmed Memon: Contribution to the concept/design; acquisition, analysis, and interpretation of data, drafting and revision of article for the content and final approval before submission.
• Mehwish Mooghal: Contribution to the concept/design; acquisition, analysis, and interpretation of data, drafting and revision of article for the content.
• Shah Wali: Contribution to the concept/design; acquisition, analysis, and interpretation of data, drafting and revision of article for the content.
• Wajiha Khan: Contribution to acquisition, analysis, and interpretation of data, drafting and revision of article for the content.
• Hina Taseen: Contribution to acquisition, analysis, and interpretation of data, drafting and revision of article for the content.
• Memuna Khan: Contribution to acquisition, analysis, and interpretation of data, drafting and revision of article for the content
• Daniyal Monis: Contribution to acquisition, analysis, and interpretation of data, drafting and revision of article for the content

## Consent

Not applicable.

## Registration of research studies

1. Name of the registry: ClinicalTrials.gov
2. Unique Identifying number or registration ID: NCT05458570
3. Hyperlink to your specific registration (must be publicly accessible and will be checked): https://clinicaltrials.gov/show/NCT05458570

## Guarantor

Dr. Wajiha Khan and Dr. Mehwish Mooghal.

## Declaration of competing interest

We do not have any conflicts of interest.
